# Progression of stenosis severity and aortopathy in adult patients with congenital aortic stenosis

**DOI:** 10.1016/j.ijcchd.2025.100646

**Published:** 2025-12-16

**Authors:** Zoë A. Keuning, Frederike Meccanici, Kevin M. Veen, Bibi Schreurs, Roland R.J. van Kimmenade, Joost P. van Melle, Monique R.M. Jongbloed, Michiel Voskuil, Berto J. Bouma, Famke Sneep, Jeroen F.A. Simons, Alexander Hirsch, Jolien W. Roos-Hesselink, Annemien E. van den Bosch

**Affiliations:** a Department of Cardiology, Cardiovascular Institute, Thorax Center, Erasmus MC, Rotterdam, the Netherlands; b Department of Cardiothoracic Surgery, Thorax Center, Erasmus MC, Rotterdam, the Netherlands; c Department of Cardiology, Radboud University Medical Center, Nijmegen, the Netherlands; d Department of Cardiology, University Medical Center Groningen, Groningen, the Netherlands; e Department of Cardiology, Leiden University Medical Center, Leiden, the Netherlands; f Department of Cardiology, University Medical Center Utrecht, Utrecht, the Netherlands; g Department of Cardiology, Academic UMC Location AMC, Amsterdam, the Netherlands; h Department of Radiology and Nuclear Medicine, Erasmus MC, Rotterdam, the Netherlands; i ERN-GUARD-Heart: European Reference Network for Rare and Low Prevalence Complex Diseases of the Heart, the Netherlands

**Keywords:** Congenital aortic stenosis, Adults, Echocardiography, Clinical course of disease

## Abstract

**Background:**

Congenital aortic stenosis (AS) is a heterogeneous disease. However, repeated data describing disease progression is limited, especially in adults. Therefore, the objective of this study is to investigate progression of peak velocity and aortic dimensions in adult congenital AS patients, identifying markers for fast progression.

**Methods:**

Adult patients (aged 18–55 years) with a native aortic valve and at least mild AS at baseline registered in the Dutch CONCOR registry between 2001 and 2019 from all six tertiary expert centers for congenital heart disease were included. Patients with severe aortic regurgitation at baseline or no available echocardiograms during follow-up were excluded. Data on ascending aortic dimensions and peak velocity changes over time were analyzed using mixed models until death, aortic valve replacement or March 1, 2023.

**Results:**

402 patients (63 % male) were included with a median age of 26 [IQR 19–41] years and peak velocity of 3.1 [IQR 2.7–3.6] m/s. Median follow-up time was 6.8 [IQR 3.8–11.6] years. Peak velocity significantly progressed over time with 0.06 ± 0.10 m/s/year (p < 0.001), independent of baseline velocity. Older age and concentric left ventricular (LV) remodeling were associated with faster progression. Mean progression of ascending aortic dimension was 0.4 ± 0.5 mm/year (p < 0.001), with faster progression in younger patients (p = 0.002). No correlation between aortic growth rate and stenosis severity progression was seen (r = 0.001, p = 0.98).

**Conclusion:**

Overall disease progression was slow in adult congenital AS patients, and independent of baseline peak velocity. Progression of stenosis should be monitored more carefully in older patients and patients with signs of concentric LV remodeling.

## Graphical abstract

**Figure:**
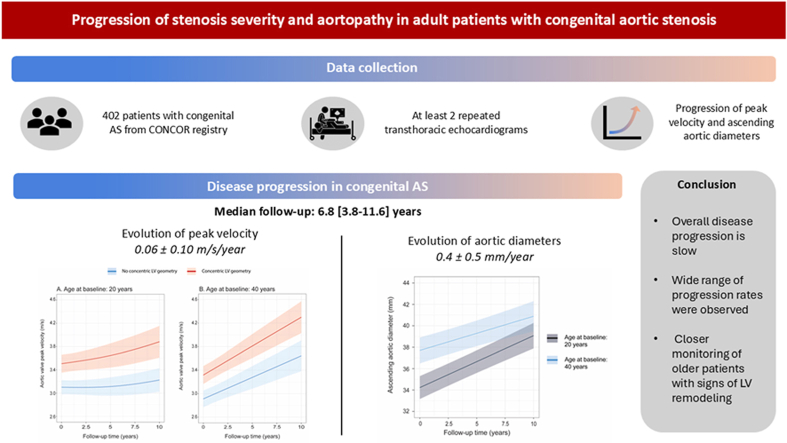

## 1 Introduction

Congenital aortic stenosis (AS) is one of the most common left-sided obstructive congenital diseases and represents 4–8 % of all congenital heart diseases (CHD) [1]. There is significant variation in the clinical course of disease. Some patients will require aortic valve intervention early in childhood [2,3], while others need aortic valve replacement (AVR) at adult age or do not need an intervention at all [4]. The progression to severe valvular dysfunction is often difficult to predict. Previous studies have identified associations between left ventricular (LV) mass, cardiovascular risk factors such as hypertension, dyslipidemia and body mass index (BMI) for fast progression of stenosis [[5], [6], [7], [8]]. However, these studies typically included children, and prospective studies describing repeated echocardiographic data on the progression of valve dysfunction in adults are limited.

Bicuspid aortic valve (BAV) is often the underlying cause of congenital AS and is strongly associated with thoracic aortic disease. The life-time risk of aortic dilatation is reported to be up to 75 % by the age of 90 [4]. The annual aortic growth rate in BAV patients varies from 0.4 to 0.8 mm/year [5,7,[9], [10], [11]]. However, most studies describe the growth rate across the entire spectrum of BAV instead of focusing on patients with congenital AS. It is debated whether dilatation is caused by hemodynamic factors, genetic factors, or a combination of both [12]. As aortic hemodynamics are believed to be influenced by the severity of valve dysfunction, differences in aortic growth for different valvular dysfunction patterns are hypothesized [13]. Therefore studying aortic growth rate in the congenital AS population is warranted.

Therefore, this study aimed to (I) describe the progression rate of stenosis severity and ascending aortic diameters in adults with congenital AS and (II) identify markers associated with disease progression.

## 2 Methods

### 2.1 Study design and population

For this multicenter observational study, all patients with congenital AS registered in the prospective CONCOR registry (Dutch registry for adult patients with CHD) between 2001 and 2019 from one of the six expert centers for CHD in the Netherlands were included. Inclusion criteria for the study were: age 18–55 years old, at least mild AS (defined as aortic valve peak velocity ≥2.5 m/s) on the echocardiogram, and availability of at least two echocardiograms during follow-up. Exclusion criteria were the presence of severe aortic regurgitation (AR) at baseline, prior AVR or on the waiting list for AVR, concomitant subvalvular or supravalvular AS, severe valvular dysfunction of the mitral valve, or concomitant complex CHD according to the ESC guidelines [14]. The study was approved by the Medical Ethical Committee (MEC 2021-0169) and performed according to the declaration of Helsinki. All patients signed informed consent for inclusion in the CONCOR registry and therefore for the use of their data for research purposes. Further informed consent was waived for this analysis.

### 2.2 Data collection

Baseline visit was defined as the first available transthoracic echocardiogram with a peak velocity ≥2.5 m/s at or after inclusion in CONCOR. Baseline characteristics were obtained from chart abstraction and consisted of clinical characteristics, cardiac surgical history, electrocardiogram data, and transthoracic echocardiogram data. During follow-up, all available transthoracic echocardiographic exams with information on either AS severity or ascending aortic diameters or both, were collected. Transesophageal echocardiograms (TEE) were rarely performed, therefore TEE data was not taken into consideration in this study. Echocardiographic exams during pregnancy were excluded. Aortic stenosis classification was based on the aortic valve peak velocity [14]. Recommendations from the American Society of Echocardiography and European Association of Cardiovascular Imaging were followed for chamber quantification [15]. Concentric LV geometry was defined as a relative wall thickness (2 ∗ LV posterior wall/LV end-diastolic dimension) > 0.42. An ascending aortic aneurysm was defined as a diameter >40 mm on any imaging modality [16]. Repeated echocardiograms were collected until death, AVR or March 1, 2023. In case of ascending aortic surgery during follow-up, measurements on aortic diameters were censored after aortic surgery.

### 2.3 Statistical analysis

Normality of data was assessed through histograms and Shapiro-Wilk test. Normally distributed continuous data was presented as mean ± standard deviation and in case data was skewed, as median [interquartile range]. Categorical variables were presented as frequencies and percentages. To assess the progression rate of aortic valve peak velocity and ascending aortic diameters per patient, a linear mixed model was used considering all repeated measurements per patient. Consequently, patients were categorized into slow, intermediate, or fast progression of stenosis. We based these tertiles on the mean aortic valve peak velocity progression rate observed per patient, categorized in three equal groups from low to high. Baseline characteristics between these three patient groups were compared using a one-way ANOVA or Kruskal-Wallis test for continuous data and a Chi-square test for categorical data. Multivariable mixed effects models were constructed using complete cases to analyze associations between baseline parameters and progression rate of ascending aortic diameter and aortic valve peak velocity. Fixed effects consisted of time in years, baseline characteristics and the interaction between time and baseline parameters. Random effects were added in the model to allow for random slopes and intercepts per patient. Natural cubic splines were added to the model if necessary to relax the assumption for linearity. Baseline characteristics included in the multivariable mixed effects models for peak velocity and diameters were based on previous literature. For peak velocity the following parameters were included: age, sex, prior valve intervention, hypertension, hyperlipidemia, aortic regurgitation severity, baseline peak velocity and LV concentric remodeling [5,6,8,17,18]. For aortic dimensions the parameters included were: age, sex, aortic coarctation, hypertension, aortic regurgitation severity and baseline peak velocity. History of hypertension was defined as diagnosis of hypertension in patient history or use of anti-hypertensive agents. Multicollinearity was checked using correlation matrix. Residual plots were checked to ascertain assumptions for the model were not violated. Sensitivity analysis was performed by inserting the mixed effects models in a Cox proportional hazard model under a joint modelling framework, to correct for dropout due to AVR or death. Two-sided p-value <0.05 was considered statistically significant. For statistical analysis SPSS (IBM Corp. Released 2021. IBM SPSS Statistics for Windows, Version 28.0. Armonk, NY: IBM Corp.) and R version 4.3.2 were used.

## 3 Results

### 3.1 Baseline characteristics

A total of 402 patients were included, with a median follow-up time of 6.8 [3.8–11.6] years. Baseline characteristics are described in Table 1. Median age at inclusion was 26 [19–41] years and 63 % of patients were male. Median aortic valve peak velocity at baseline was 3.1 [2.7–3.6] m/s and 143 patients (35.8 %) had moderate AR. History of hypertension was present in 78 patients (19.4 %) and 18 patients had hypercholesterolemia (4.5 %). Nine patients were known with Turner syndrome (2.2 %) and one patient was known with a connective tissue disorder (Ehlers-Danlos syndrome; 0.2 %). LV systolic function was impaired in 12 patients (3.1 %) at baseline.

**Table 1:** Baseline characteristics.

	All patients N = 402	Slow progression N = 134	Intermediate progression N = 134	Fast progression N = 134	P-value
**Patient characteristics**					
Age, years	26 [19–41]	22 [19–30]	26 [19–41]	35 [24–45]	<0.001
Sex, male	254 (63.2)	90 (67.2)	82 (61.2)	82 (61.2)	0.50
Body surface area, m^2^	1.9 ± 0.2	1.9 ± 0.2	1.9 ± 0.2	1.9 ± 0.2	0.56
Bicuspid aortic valve	370 (93.0)	123 (92.5)	121 (92.4)	126 (94.0)	0.84
Systolic blood pressure, mmHg	124 [115–136]	125 [115–136]	121 [115–135]	125 [115–140]	0.57
Diastolic blood pressure, mmHg	76 [70–84]	73 [68–80]	75 [70–84]	80 [70–87]	0.01
Smoking					0.15
Current	54 (16.5)	15 (13.3)	20 (19.0)	19 (17.4)	
Past	53 (16.2)	12 (10.6)	21 (20.0)	20 (18.3)	
NYHA class ≥2	22 (5.8)	8 (6.3)	6 (4.9)	8 (6.2)	0.88
Turner syndrome	9 (2.2)	4 (3.0)	5 (3.7)	0 (0)	0.09
Concomitant CHD	47 (11.7)	20 (14.9)	12 (9.0)	15 (11.2)	0.31
Hypertension	78 (19.4)	22 (16.4)	18 (13.4)	38 (28.4)	0.01
Hyperlipidemia	18 (4.5)	3 (2.2)	5 (3.8)	10 (7.6)	0.10
Diabetes mellitus	4 (1.0)	0 (0)	4 (3.0)	0 (0)	0.02
Aortic coarctation	82 (19.9)	30 (22.4)	27 (20.1)	25 (18.7)	0.75
Treated	72 (87.8)	26 (86.7)	23 (85.2)	23 (92.0)	0.73
Ascending aortic aneurysm	92 (23.1)	26 (19.4)	36 (26.9)	31 (23.1)	0.35
Treated	9 (9.7)	4 (15.4)	3 (8.3)	2 (6.5)	0.49
Prior AV intervention	93 (23.1)	30 (22.4)	35 (26.1)	28 (20.9)	0.58
**Echocardiography**					
Impaired LV systolic function	12 (3.1)	5 (4.0)	2 (1.5)	5 (3.8)	0.43
LV end-diastolic dimension, mm	51 ± 6	52 ± 7	52 ± 6	49 ± 6	<0.001
LV end-systolic dimension, mm	31 ± 6	32 ± 5	32 ± 5	30 ± 6	<0.001
LV mass index, g/m^2^	92 [78–111]	90 [77–110]	94 [79–120]	91 [78–110]	0.32
LV concentric remodeling	98 (28.8)	18 (15.7)	29 (24.6)	51 (47.7)	<0.001
E/A ratio	1.5 [1.2–1.9]	1.6 [1.4–2.0]	1.6 [1.3–1.9]	1.4 [0.9–1.9]	0.02
AV peak velocity, m/s	3.1 [2.7–3.6]	2.9 [2.7–3.3]	3.1 [2.7–3.6]	3.3 [2.8–3.8]	<0.001
Aortic regurgitation					0.29
None-mild	256 (64.2)	85 (63.9)	80 (59.7)	91 (68.9)	
Moderate	143 (35.8)	48 (36.1)	54 (40.3)	41 (31.1)	
Ascending aortic diameter, mm	36 ± 7	35 ± 7	37 ± 7	36 ± 7	0.04
**Electrocardiography**					
Sinus rhythm	386 (98.5)	128 (97.7)	130 (99.2)	128 (98.5)	0.60
Heart rate, bpm	69 ± 12	67 ± 13	68 ± 12	70 ± 13	0.16

AV, aortic valve; CHD, congenital heart disease; LV, left ventricle; NYHA, New York Heart Association.

Data is presented as number (percentage), mean ± standard deviation or median [25th-75th percentile].

### 3.2 Progression of stenosis severity

For the progression of aortic valve peak velocity, 2708 repeated echocardiograms were analyzed with a median of 6 [3–9] follow-up echocardiograms per patient. The mean progression rate was 0.06 ± 0.10 m/s per year (p < 0.001). Baseline characteristics stratified according to these tertiles (slow progression: <0.03 m/s/year, intermediate progression: 0.03–0.08 m/s/year and fast progression: >0.08 m/s/year) are displayed in Table 1. Patients with faster progression rates were significantly older compared to those with slower progression (22 [19–30] vs. 26 [19–41] vs. 35 [24–45] years, p < 0.001). Moreover, patients with faster progression had significantly more often a history of hypertension (16.4 % vs. 13.4 % vs. 28.4 %, p < 0.001). Types of LV remodeling significantly differed between the three patient groups, with a higher percentage of LV concentric geometry (15.7 % vs. 24.7 % vs. 47.7 %) in patients with fast progression (p < 0.001).

**Fig. 1:**
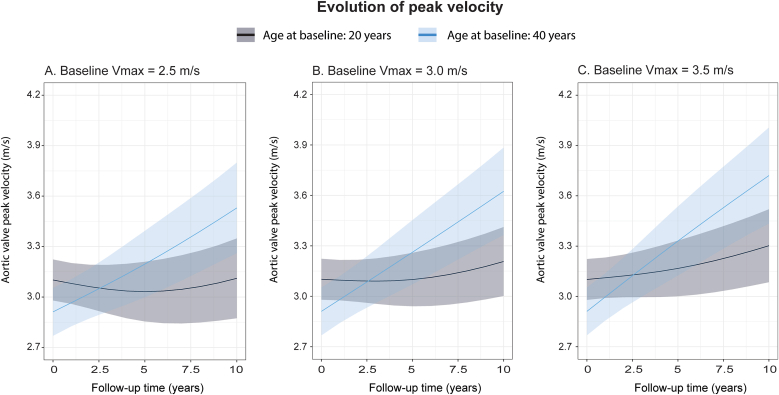
Effect plots of aortic valve peak velocity evolution with 95 % confidence interval based on the multivariable mixed model, stratified per age. Progression is displayed according to varying baseline aortic valve peak velocity (present in the model only as interaction with time).

**Fig. 2:**
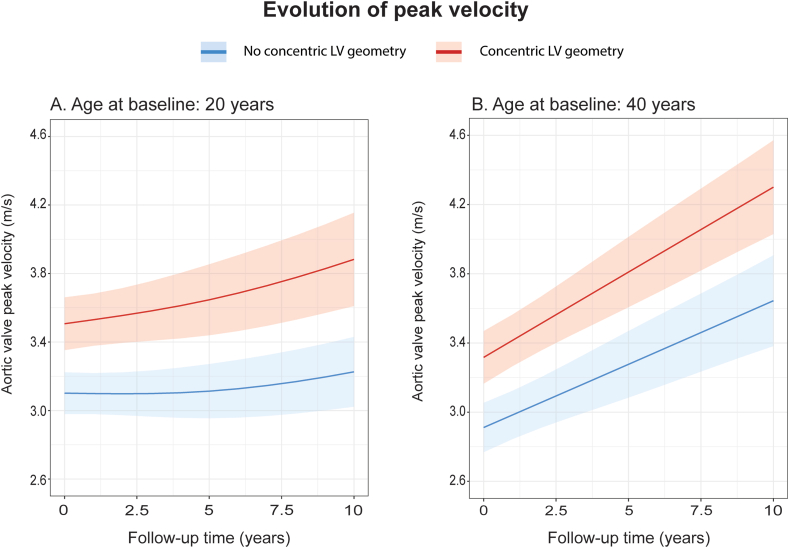
Effect plots of evolution of aortic valve peak velocity with 95 % confidence interval based on the multivariable mixed model, stratified based on LV geometry. A) Age at baseline 20 years. B) Age at baseline 40 years.

The mixed effects model for peak velocity was fitted with two splines in the random and fixed effects (Supplementary Table S1). Older age at baseline was independently associated with faster progression of stenosis during the entire follow-up irrespective of baseline peak velocity (time spline 1: p < 0.001, time spline 2: p = 0.03). Effect plots of the multivariable model comparing an average 20-year with a 40-year-old patient, shows the differences in progression, with younger patients showing almost no progression during the first years of follow-up (Fig. 1). Moreover, the presence of concentric LV geometry was significantly associated with faster progression in the first spline (Fig. 2). Progression rates were not influenced by sex, prior valve intervention, moderate aortic regurgitation, history of hypertension or hypercholesterolemia. Progression of aortic valve peak velocity in the first spline was significantly correlated with progression in the second spline (r = 0.62, p < 0.001).

### 3.3 Progression of ascending aortic diameters

**Fig. 3:**
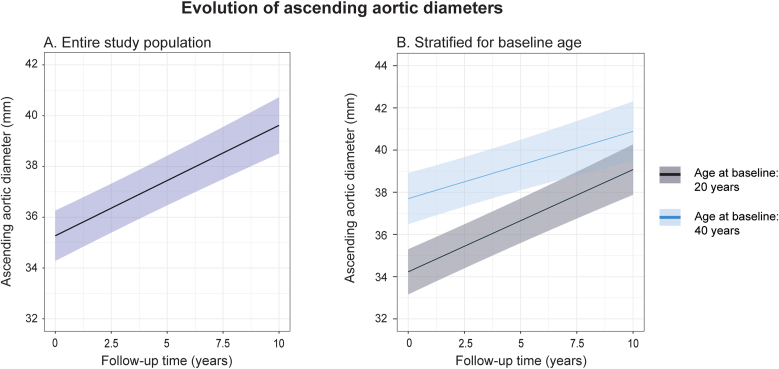
Effect plots of evolution of ascending aortic diameters. A) For the entire study population. B) Stratified for age at baseline.

In the mixed effects model for ascending aortic dimension, the model was fitted without splines in the random and fixed effects (Supplementary Table S2). The progression of ascending aortic diameters was assessed in 2074 repeated echocardiograms in 386 patients, with a median of 4 [2–7] echocardiograms per patient. Ascending aortic diameters significantly increased over time, with a mean progression rate of 0.4 ± 0.5 mm/year (p < 0.001). Older patients showed significantly slower progression of aortic diameters (Fig. 3). Sex, aortic coarctation, history of hypertension, baseline aortic valve peak velocity and moderate aortic regurgitation were not significantly associated with aortic growth rate (Supplementary Table S2). Aortic growth was not correlated with the progression of aortic valve peak velocity (r = 0.001, p = 0.98).

### 3.4 Sensitivity analysis

Sensitivity analysis was performed to correct for drop-out due to AVR or death by modeling the longitudinal evolution of peak velocity into a joint model. Some changes were observed in the effect size and standard errors within the joint model compared to the linear mixed model, but the direction and significance of the effects did not change (Supplementary Table S3). The joint model, combining the mixed model for ascending aortic diameters with the survival model, also showed no changes in direction and significance of the effects on aortic growth rate (Supplementary Table S4).

## 4 Discussion

In this study, disease progression was evaluated in a large population of young adults with congenital valvular AS, assessing the progression of stenosis severity and ascending aortic dimensions. Over a median follow-up period of 7 years, there was a statistically significant increase in both aortic valve stenosis severity and ascending aortic diameters. Although overall the rate of progression of aortic valve peak velocity was low, some patients did show higher progression rates. Faster progression of stenosis was associated with older age and the presence of concentric LV geometry, whereas cardiovascular risk factors (hypertension and hyperlipidemia) and baseline AS severity were not found to be significantly associated. In contrast, for ascending aortic diameters, higher age was associated with a lower aortic growth rate.

### 4.1 Progression of stenosis severity

Data on the progression of aortic valve peak velocity in adult patients with congenital AS are limited. Previous studies described progression rates between 0.07 and 0.18 m/s per year [6,8,[18], [19], [20], [21]] or even no progression over time [7]. In our study evaluating >2700 echocardiograms, the progression rate was also low, with an average yearly increase of 0.06 m/s. However, progression rates did vary between patients, as the rates ranged to a maximum of 0.3 m/s/year. It is of great importance to distinguish fast-progressive from slow-progressive patients with congenital AS to either prolong or shorten follow-up intervals accordingly and minimize health care burden as much as possible.

Previous studies described a significant influence of cardiovascular risk factors on the progression of AS in BAV patients [6,8,18]. In our study, however, associations with history of hypertension and hypercholesterolemia were not observed in the multivariable model. A possible explanation for this discrepancy is the difference in age in the studied populations (median of 29 years vs. median of 50–60 years). Two prior studies that did describe progression in the young adult congenital AS population also found no association of cardiac risk factors with progression [7,21]. It has been previously noted that the pathogenesis of aortic valve calcification might differ between younger and older patients [22]. Possibly, other factors are more important as prognostic markers in the young adult congenital AS population. In multivariable analysis, age remained associated with rapid progression. In young patients, almost no progression was seen in the first 5 years of follow-up, irrespective of baseline AS severity. However, as time progressed and patients aged, there was a clear increase visible in peak velocity. The progression of degenerative AS follows similar pathways as atherosclerosis, with an active role for lipid infiltration and inflammation [23], which have also been described in patients with a stenotic BAV [24]. It is known that age significantly influences the development of aortic valve calcification [25], and that with aortic valve calcification progression, pressure gradients over the valve increases [26]. It can therefore be hypothesized with the aging patient, more calcification causes faster progression. Moreover, it is possible that longer exposure to shear stress due to the congenital abnormal valve influences disease progression in older adults.

Remarkably, the presence of concentric LV geometry was also associated with faster progression of stenosis in our study. This is in line with previous studies in congenital AS patients, which described higher LV mass index in patients with rapid progression of stenosis [7,17]. LV remodeling is believed to be an adaptive response to the chronic LV pressure overload caused by AS [27]. However, although our results indicate a relation between LV remodeling and stenosis progression, no association with hypertension or baseline peak velocity was observed. This further strengthens the importance of monitoring LV remodeling besides factors that influence the increasing afterload. Previous research also indicated that the hypertrophic response of the LV is not necessarily associated with the severity of stenosis [28,29], and the precise cause for LV remodeling in AS patients is still debated. It has been suggested that it is not only an adaptive response to maintain cardiac output, but is also influenced by genetic and neurohormonal factors [30]. Concentric LV remodeling and hypertrophy have been associated with worse clinical outcomes [31,32]. Our study results underline the negative influence of LV remodeling on disease progression. It can be hypothesized that the presence of concentric LV geometry characterizes a more severe phenotype of the disease, which should be given more emphasis in clinical decision-making.

### 4.2 Progression of aortic diameters

Currently available research on aortic growth rates has been focused on the entire spectrum of BAV patients, while studies focusing solely on adults with stenosis are scarce. In our study a mean progression rate of 0.4 mm/year was observed, which is in line with earlier studies [5,11,33,34]. Interestingly, we did not find a significant influence of severity of aortic regurgitation on growth rate of the aorta in our study. The only marker that did significantly influence the progression of aortic diameters was age, with a higher progression rate observed in younger patients. Although this finding has been described previously in older cohorts of BAV patients [5,11], this association was not previously seen in the young congenital AS population [7,20]. It can be hypothesized that this is due to selection bias, as we only included patients without prior AVR. Consequently, the older patients included in our study reflect the patients who aged without needing an intervention, probably marking those with a milder phenotype of disease.

In our study, no influence of AS severity was observed. Moreover, the progression rate of AS was not associated with the progression rate of aortic diameters. These findings further support the theory that beside hemodynamic factors, genetic predisposition also plays an important role in developing aortic dilatation.

### 4.3 Clinical implications

The management of asymptomatic patients with AS remains an important challenge for clinicians. Lifelong regular monitoring is recommended and diagnostic imaging is performed frequently to ensure most optimal timing for intervention [14]. However, the appropriate monitoring intervals and the ideal timing for intervention is still a topic of debate. Although the progression rate of aortic valve peak velocity and aortic growth were low in our study, it varied significantly among patients. Previous studies identified modifiable risk factors such as hypertension, diabetes, and BMI as predictors for fast progression of stenosis, but this was not observed in our study population of young adult patients. In our study, only increasing age and LV remodeling were associated with progression, both non-modifiable risk factors. Therefore, close monitoring the presence of LV remodeling during outpatient visits is necessary, to be able to monitor these patients with a higher chance of fast progression. On the other hand, it might be considered to extend follow-up intervals in selected low-risk young adults without any evidence for LV remodeling. Future research should also include non-imaging parameters, such as blood biomarkers (e.g. NT-pro BNP), which may provide important prognostic information [35]. Current guidelines describe a progression rate of ≥0.3 m/s/year in asymptomatic patients with normal LV function as a risk factor in which valve intervention could be considered (class IIa) [14]. Based on the overall low progression rates found in our study population, it can however be questioned whether this cut-off value for progression rate of 0.3 m/s/year is not too high, as it was not often reached in our patient population.

## 5 Limitations

There are a few limitations to this study. First, all patients were included from tertiary care centers with expertise in adult CHD and only included patients that consented to participation in the CONCOR registry, causing potential selection bias. This could influence the generalizability of our study results. Second, imputation was not performed, and multivariable mixed models were performed on cases without missing data points in the included variables, which resulted in fewer cases available to assess associations with progression rates. Moreover, due to a high extent of missing data on BAV morphology this parameter could not be considered in the multivariable model. Aortic diameters are routinely assessed with echocardiography in clinical practice and therefore used to evaluate aortic growth in this study. Assessing aortic growth on CT or MRI would also be of great interest and possibly even more representative, however CT/MRI was not performed regularly during the study period. Lastly, patients were censored in our study in case of AVR, which could have influenced the linear mixed models. However, to correct for survival bias, joint models were constructed, which did not show different results regarding the direction or significance of effects.

## 6 Conclusion

In this young adult congenital AS population, overall disease progression was slow. However, a wide variety was seen in progression rates of stenosis severity, which underlines the importance of distinguishing patients with slow and fast progressive disease. Our data suggest that older patients with signs of LV remodeling should be monitored more closely, while in low-risk young patients with no signs of remodeling, it could be considered to extend follow-up intervals up to 5 years.

## CRediT authorship contribution statement

**Zoë A. Keuning:** Writing – review & editing, Writing – original draft, Visualization, Validation, Resources, Project administration, Methodology, Investigation, Formal analysis, Data curation, Conceptualization. **Frederike Meccanici:** Writing – review & editing, Validation, Project administration, Methodology, Investigation, Data curation, Conceptualization. **Kevin M. Veen:** Writing – review & editing, Visualization, Validation, Methodology, Formal analysis. **Bibi Schreurs:** Writing – review & editing, Validation, Resources, Investigation, Data curation. **Roland R.J. van Kimmenade:** Writing – review & editing, Validation, Resources, Investigation. **Joost P. van Melle:** Writing – review & editing, Validation, Resources, Investigation. **Monique R.M. Jongbloed:** Writing – review & editing, Validation, Resources, Investigation. **Michiel Voskuil:** Writing – review & editing, Validation, Resources, Investigation. **Berto J. Bouma:** Writing – review & editing, Validation, Resources, Investigation. **Famke Sneep:** Writing – review & editing, Validation, Investigation, Data curation. **Jeroen F.A. Simons:** Writing – review & editing, Validation, Investigation, Data curation. **Alexander Hirsch:** Writing – review & editing, Validation, Supervision, Investigation. **Jolien W. Roos-Hesselink:** Writing – review & editing, Validation, Supervision, Resources, Methodology, Investigation, Conceptualization. **Annemien E. van den Bosch:** Writing – review & editing, Validation, Supervision, Resources, Methodology, Investigation, Conceptualization.

## Disclosures

J.W. Roos-Hesselink is an Editorial Board Member of the International Journal of Cardiology Congenital Heart Disease and had no involvement in the Journal's evaluation of this article.

## Sources of funding

We acknowledge the support from the Netherlands Cardiovascular Research initiative: An initiative with support of the Dutch 10.13039/100002129Heart Foundation, the Netherlands and Hartekind, CVON2019-002 OUTREACH.

## Declaration of competing interest

The authors declare the following financial interests/personal relationships which may be considered as potential competing interests:A.E. van den Bosch reports financial support was provided by Dutch Heart Foundation. Other authors declare that they have no known competing financial interests or personal relationships that could have appeared to influence the work reported in this paper.
