# Supplementary material for: Progression of stenosis severity and aortopathy in adult patients with congenital aortic stenosis
Source: Int J Cardiol Congenit Heart Dis. 2025 Dec 16;23:100646. doi: 10.1016/j.ijcchd.2025.100646 (PMC12828805; doi:10.1016/j.ijcchd.2025.100646)
Supplement: Multimedia component 1 [file mmc1.docx]

**Supplemental material**

**Supplementary table 1.** Multivariable linear mixed-effects model for aortic valve peak velocity progression over time using complete cases. The model was fitted with a spline function containing 1 knot in the fixed and random effects.

Number of patients: 336.

Number of repeated measurements: 2357.

|  | **Estimate (SE)** | **P-value** |
| --- | --- | --- |
| **Characteristic** |  |  |
| Intercept | 3.29 (0.11) | <0.001 |
| Time spline 1 | -1.94 (0.72) | 0.01 |
| Time spline 2 | -0.37 (1.03) | 0.72 |
| Age, years | -0.01 (0.00) | 0.004 |
| Sex, female | 0.05 (0.07) | 0.42 |
| Prior valvular intervention | 0.10 (0.08) | 0.20 |
| History of hypertension | -0.08 (0.09) | 0.36 |
| History of hyperlipidemia | -0.04 (0.16) | 0.82 |
| Aortic regurgitation, moderate | 0.12 (0.07) | 0.07 |
| Concentric LV geometry | 0.41 (0.07) | <0.001 |
| **Interaction with time** |  |  |
| Age: time spline 1 | 0.06 (0.01) | <0.001 |
| Age: time spline 2 | 0.04 (0.02) | 0.03 |
| Sex, female: time spline1 | 0.18 (0.20) | 0.36 |
| Sex, female: time spline 2 | 0.07 (0.25) | 0.79 |
| Prior valvular intervention: time spline 1 | -0.06 (0.22) | 0.78 |
| Prior valvular intervention: time spline 2 | 0.09 (0.26) | 0.73 |
| History of hypertension: time spline 1 | 0.28 (0.29) | 0.34 |
| History of hypertension: time spline 2 | 0.23 (0.44) | 0.60 |
| History of hyperlipidemia: time spline 1 | 0.82 (0.52) | 0.11 |
| History of hyperlipidemia: time spline 2 | 1.04 (0.83) | 0.22 |
| Aortic regurgitation, moderate: time spline 1 | -0.19 (0.20) | 0.33 |
| Aortic regurgitation, moderate: time spline 2 | 0.07 (0.25) | 0.77 |
| Concentric LV geometry: time spline 1 | 0.49 (0.22) | 0.03 |
| Concentric LV geometry: time spline 2 | 0.41 (0.29) | 0.16 |
| Baseline AV peak velocity: time spline 1 | 0.34 (0.19) | 0.08 |
| Baseline AV peak velocity: time spline 2 | 0.07 (0.27) | 0.81 |

**Supplementary table 2.** Multivariable linear mixed-effects model for ascending aortic diameter progression over time using complete cases. The model was fitted with random intercepts and random slopes.

Number of patients: 384

Number of repeated measurements: 2070

|  | **Estimate (SE)** | **P-value** |
| --- | --- | --- |
| **Characteristics** |  |  |
| Intercept | 25.19 (2.07) | <0.001 |
| Time, years | 0.59 (0.20) | 0.003 |
| Age, years | 0.17 (0.03) | <0.001 |
| Sex, female | -1.86 (0.63) | 0.004 |
| History of coarctation | -2.98 (0.83) | <0.001 |
| History of hypertension | -0.35 (0.87) | 0.69 |
| Aortic regurgitation, moderate | 1.10 (0.65) | 0.09 |
| Baseline AV peak velocity | 1.80 (0.55) | 0.001 |
| **Interaction with time** |  |  |
| Age:time | -0.01 (0.00) | 0.002 |
| Sex:time | -0.02 (0.05) | 0.71 |
| History of coarctation:time | -0.05 (0.07) | 0.51 |
| History of hypertension:time | -0.05 (0.08) | 0.52 |
| Aortic regurgitation, moderate:time | -0.03 (0.05) | 0.61 |
| Baseline AV peak velocity:time | 0.02 (0.05) | 0.70 |

|  | **Mixed model** | | **Joint model** | |
| --- | --- | --- | --- | --- |
|  | **Estimate** | **P-value** | **Estimate** | **P-value** |
| **Characteristic** |  |  |  |  |
| Intercept | 3.29 | <0.001 | 3.28 | <0.001 |
| Time spline 1 | -1.94 | 0.01 | -0.24 | 0.77 |
| Time spline 2 | -0.37 | 0.72 | -0.79 | 0.30 |
| Age, years | -0.01 | 0.004 | -0.01 | 0.002 |
| Sex, female | 0.05 | 0.42 | 0.05 | 0.38 |
| Prior valvular intervention | 0.10 | 0.20 | 0.10 | 0.20 |
| History of hypertension | -0.08 | 0.36 | -0.09 | 0.32 |
| History of hyperlipidemia | -0.04 | 0.82 | -0.07 | 0.66 |
| Aortic regurgitation, moderate | 0.12 | 0.07 | 0.12 | 0.05 |
| Concentric LV geometry | 0.41 | <0.001 | 0.40 | <0.001 |
| **Interaction with time** |  |  |  |  |
| Age: time spline 1 | 0.06 | <0.001 | 0.05 | <0.001 |
| Age: time spline 2 | 0.04 | 0.03 | 0.04 | <0.001 |
| Sex, female: time spline1 | 0.18 | 0.36 | 0.21 | 0.20 |
| Sex, female: time spline 2 | 0.07 | 0.79 | 0.08 | 0.65 |
| Prior valvular intervention: time spline 1 | -0.06 | 0.78 | 0.03 | 0.88 |
| Prior valvular intervention: time spline 2 | 0.09 | 0.73 | 0.09 | 0.62 |
| History of hypertension: time spline 1 | 0.28 | 0.34 | 0.22 | 0.37 |
| History of hypertension: time spline 2 | 0.23 | 0.60 | 0.13 | 0.71 |
| History of hyperlipidemia: time spline 1 | 0.82 | 0.11 | 0.73 | 0.10 |
| History of hyperlipidemia: time spline 2 | 1.04 | 0.22 | 0.52 | 0.41 |
| Aortic regurgitation, moderate: time spline 1 | -0.19 | 0.33 | -0.17 | 0.31 |
| Aortic regurgitation, moderate: time spline 2 | 0.07 | 0.77 | -0.02 | 0.95 |
| Concentric LV geometry: time spline 1 | 0.49 | 0.03 | 0.75 | <0.001 |
| Concentric LV geometry: time spline 2 | 0.41 | 0.16 | 0.30 | 0.13 |
| Baseline AV peak velocity: time spline 1 | 0.34 | 0.08 | -0.18 | 0.46 |
| Baseline AV peak velocity: time spline 2 | 0.07 | 0.81 | 0.18 | 0.39 |

**Supplementary table 3.** Estimates of the multivariable mixed model for aortic valve peak velocity compared to the longitudinal outcome of the joint model. No differences of direction or significance of effect were observed.

**Supplementary table 4.** Estimates of the multivariable mixed model for ascending aortic diameter compared to the longitudinal outcome of the joint model. No differences of direction or significance of effect were observed.

|  | **Mixed model** | | **Joint model** | |
| --- | --- | --- | --- | --- |
|  | **Estimate** | **P-value** | **Estimate** | **P-value** |
| **Characteristics** |  |  |  |  |
| Intercept | 25.19 | <0.001 | 25.04 | <0.001 |
| Time, years | 0.59 | 0.003 | 0.53 | 0.006 |
| Age, years | 0.17 | <0.001 | 0.18 | <0.001 |
| Sex, female | -1.86 | 0.004 | -1.87 | 0.002 |
| History of coarctation | -2.98 | <0.001 | -3.01 | <0.001 |
| History of hypertension | -0.35 | 0.69 | -0.51 | 0.53 |
| Aortic regurgitation, moderate | 1.10 | 0.09 | 1.13 | 0.07 |
| Baseline AV peak velocity, m/s | 1.80 | 0.001 | 1.84 | <0.001 |
| **Interaction with time** |  |  |  |  |
| Age:time | -0.01 | 0.002 | -0.01 | <0.001 |
| Sex:time | -0.02 | 0.71 | -0.02 | 0.62 |
| History of coarctation:time | -0.05 | 0.51 | -0.04 | 0.62 |
| History of hypertension:time | -0.05 | 0.52 | -0.01 | 0.85 |
| Aortic regurgitation, moderate:time | -0.03 | 0.61 | -0.04 | 0.43 |
| Baseline AV peak velocity:time | 0.02 | 0.70 | 0.03 | 0.48 |
